# Successful preoperative esophageal cleansing for achalasia with esophageal dilatation using the drainage method and gel immersion

**DOI:** 10.1055/a-2615-6089

**Published:** 2025-05-26

**Authors:** Satoshi Abiko, Haruhiro Inoue, Kei Ushikubo, Kazuki Yamamoto, Yohei Nishikawa, Ippei Tanaka, Naoya Sakamoto

**Affiliations:** 1 378609 Digestive Diseases Center, Showa University Koto Toyosu Hospital, Koto, Japan; 2 163693 Gastroenterology and Hepatology, Hokkaido University Hospital, Sapporo, Japan

During preoperative esophageal cleansing for achalasia with esophageal dilatation, passing through the esophagogastric junction (EGJ) and maintaining the lower esophageal sphincter in an open position is a critical step to facilitate the drainage of large amounts of food residue into the stomach. This residue is often too voluminous to be aspirated directly. We have named this technique the drainage method. However, the presence of a large amount of food residue may obscure the endoscopic field, making EGJ passage difficult and increasing the risk of serious complications such as esophageal perforation or aspiration pneumonia.

Gel immersion using a transparent gel (VISCOCLEAR; Otsuka Pharmaceutical Factory, Inc., Tokushima, Japan) has been proposed as an effective approach to enhance endoscopic visualization 1 2 3 . We describe a case of successful preoperative esophageal cleansing for achalasia with esophageal dilatation using a combination of the drainage method and gel immersion ( Video 1 ).

Demonstration of preoperative esophageal cleansing for achalasia with esophageal dilatation using the drainage method combined with gel immersion in a 72-year-old man.Video 1

The procedure was performed in a 72-year-old man with achalasia and marked esophageal dilatation ( Fig. 1 **a** ). To prevent aspiration pneumonia, the procedure began with the patient’s head elevated at approximately 70°. Initial suctioning of the food residue was attempted but proved unsuccessful due to the amount of the material. As a result, the drainage method was attempted ( Fig. 1 **b** ). Efforts to reach the EGJ while maintaining visibility using water were hindered by the large volume of food residue. To improve visualization, gel was introduced, which successfully clarified the endoscopic view ( Fig. 2 **a** ) and allowed identification of the EGJ ( Fig. 2 **b** ). After traversing the EGJ, the lower esophageal sphincter was dilated, and the procedure was continued in the esophagus. A combination of water and CO₂ insufflation was then used to promote further drainage of the food residue into the stomach. The esophageal cleansing procedure was successfully completed ( Fig. 1 **c** ).

**Fig. 1:**
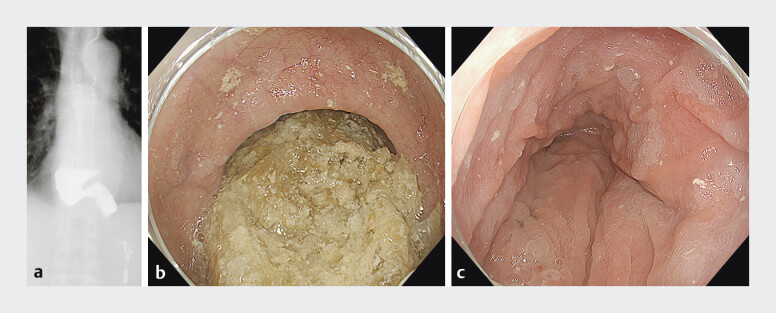
Barium examination performed before and after preoperative esophageal cleansing for achalasia with esophageal dilatation using the drainage method and gel immersion. **a** Barium examination in a patient with achalasia and esophageal dilation. **b** Suctioning of food residue was attempted but proved unsuccessful due to the large volume and size of the residue; the drainage method was therefore employed. **c** Preoperative esophageal cleansing for achalasia with esophageal dilatation was successfully completed.

**Fig. 2:**
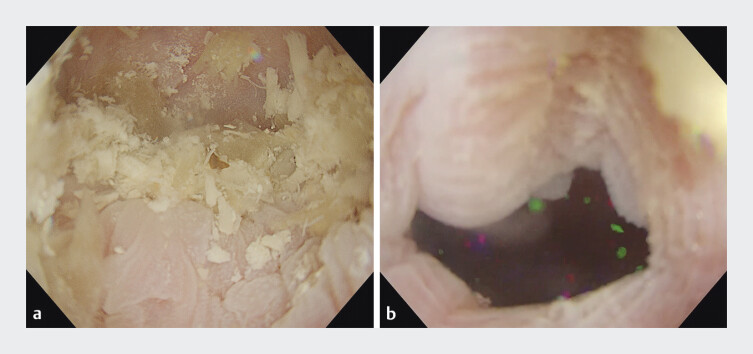
Drainage method with gel immersion. **a** Gel was used to achieve a clear endoscopic view. **b** The gastroesophageal junction was successfully identified.

Preoperative esophageal cleansing for achalasia with esophageal dilatation using the drainage method in conjunction with gel immersion may be an effective technique that may reduce the risk of severe complications.

Endoscopy_UCTN_Code_TTT_1AO_2AD

## Notes

Endoscopy E-Videos https://eref.thieme.de/e-videos
*E-Videos* is an open access online section of the journal *Endoscopy* , reporting on interesting cases and new techniques in gastroenterological endoscopy. All papers include a high-quality video and are published with a Creative Commons CC-BY license. Endoscopy E-Videos qualify for HINARI discounts and waivers and eligibility is automatically checked during the submission process. We grant 100% waivers to articles whose corresponding authors are based in Group A countries and 50% waivers to those who are based in Group B countries as classified by Research4Life (see: https://www.research4life.org/access/eligibility/ ). This section has its own submission website at https://mc.manuscriptcentral.com/e-videos .
